# The growing armamentarium of image-guided tumor ablation in interventional oncology

**DOI:** 10.1093/radadv/umaf033

**Published:** 2025-09-11

**Authors:** Jung H Yun, Arian Mansur, Elahe Abbaspour, Seyed Sina Zakavi, Callum S Newton, David C Madoff, David Woodrum, Christos Georgiades, Paul Shyn, Aria F Olumi, Raul N Uppot, Sanjeeva Kalva, Nima Kokabi, Ripal Gandhi, Peiman Habibollahi, Nariman Nezami

**Affiliations:** Department of Diagnostic and Interventional Radiology, Jefferson Einstein Philadelphia Hospital, Philadelphia, PA, 19141, United States; Division of Vascular and Interventional Radiology, Department of Radiology, Harvard Medical School, Beth Israel Deaconess Medical Center, Boston, MA, 02215, United States; Harvard Medical School, Boston, MA, 02115, United States; Department of Radiology, Ghaem International Hospital, Rasht, 6541743897, Iran; Clinical Research Development Unit of Tabriz Valiasr Hospital, Tabriz University of Medical Sciences, Tabriz, 5165665931, Iran; Biomedical Graduate Education, Georgetown University, Washington, DC, 20057, United States; Department of Radiology and Biomedical Imaging, Section of Interventional Radiology, Yale University School of Medicine, New Haven, CT, 06510, United States; Department of Medicine, Section of Medical Oncology, Yale University School of Medicine, New Haven, CT, 06510, United States; Department of Surgery, Section of Surgical Oncology, Yale University School of Medicine, New Haven, CT, 06510, United States; Department of Radiology, Mayo Clinic, Rochester, MN, 55905, United States; Division of Vascular and Interventional Radiology, Russell H. Morgan Department of Radiology and Radiological Science, The Johns Hopkins School of Medicine, Baltimore, MD, 21205, United States; Division of Abdominal Imaging and Intervention, Department of Radiology, Harvard Medical School, Brigham and Women’s Hospital, Boston, MA, 02115, United States; Department of Urologic Surgery, Beth Israel Deaconess Medical Center, Boston, MA, 02215, United States; Division of Interventional Radiology, Massachusetts General Hospital, Boston, MA, 02115, United States; Department of Radiology, UT Southwestern Medical Center, Dallas, TX, 75390, United States; Department of Radiology, University of North Carolina School of Medicine, Chapel Hill, NC, 27599, United States; Miami Cardiac and Vascular Institute, Miami, FL, 33176, United States; Department of Interventional Radiology, University of Texas MD Anderson Cancer Center, Houston, TX, 77030, United States; Division of Interventional Radiology, Department of Radiology, The Georgetown University Medical Center, Washington, DC, 20057, United States; Georgetown University School of Medicine, Washington, DC, 20007, United States; Lombardi Comprehensive Cancer Center, Washington, DC, 20007, United States

**Keywords:** interventional oncology, ablation, non-thermal, non-invasive, irreversible electroporation, pulsed electric field, HIFU, histotripsy

## Abstract

Minimally invasive image-guided tumor ablation techniques have been established as safe, effective methods to treat a variety of unresectable soft tissue tumors. Standard thermal ablation methods include radiofrequency ablation, microwave ablation, and cryoablation. However, newer non-thermal and/or non-invasive ablation techniques are now available as alternative options to treat soft tissue tumors, particularly those that are near critical structures or otherwise susceptible to thermal energy sink effects. The 4 types of emerging ablation techniques discussed in this review are as follows: irreversible electroporation, pulsed electric field, high-intensity focused ultrasound, and histotripsy. While the clinical trials evaluating the safety and efficacy of these ablation techniques are in their early stages, initial results are promising in the treatment of various cancers at different stages. These include potential synergistic effects when combined with chemotherapy and immunotherapy.

**Abbreviations**

RFA = Radiofrequency ablation; MWA = Microwave ablation; IO = Interventional oncology; IRE = Irreversible electroporation; PEF = Pulsed electric field; HIFU = High-intensity focused ultrasound; FDA = Food and Drug Administration; CRLM = Colorectal liver metastases; HCC = Hepatocellular carcinoma; SOC = Standard of care

**Summary**

Emerging non-invasive or non-thermal image-guided tumor ablation techniques include irreversible electroporation, pulsed electric field, high-intensity focused ultrasound, and histotripsy, which are redefining the scope of interventional oncology.

Essentials

- Traditional image-guided thermal ablation methods are limited by the presence of nearby thermal energy sink effects and temperature-sensitive structures.
- Non-thermal ablation techniques allow for the treatment of tumors that are surgically unresectable and near critical structures.
- Emerging ablation techniques, such as high-intensity focused ultrasound and histotripsy, provide the added benefit of a non-invasive approach.
- Early clinical studies demonstrate promising results regarding the safety and efficacy of these techniques in treating various cancers in different stages.

## Introduction

Minimally invasive image-guided tumor ablation techniques have been established as safe, effective methods to treat a variety of solid tumors.^1^ The most common percutaneous tumor ablation methods are positive thermal ablation techniques, including radiofrequency ablation (RFA) and microwave ablation (MWA), and less commonly laser ablation, which achieve coagulative necrosis and tumor destruction by targeting tissue temperature of at least 60 °C.^1^^,^^2^ In contrast, cryoablation, another thermal ablation modality, applies negative thermal energy by delivering freezing temperatures to destroy tumor tissue. While thermal ablation remains widely used as the standard for many tumor treatments in interventional oncology (IO), it carries the risks of thermal damage to adjacent critical structures, incomplete tumor eradication in borderline-sized tumors, and thermal energy sink effects that can reduce treatment efficacy in highly vascular tumors.^1–3^ These challenges have led to increased interest in non-thermal ablation techniques.

Irreversible electroporation (IRE) has emerged as a non-thermal ablation technique to treat solid organ tumors, including those in the liver, pancreas, prostate, and kidney. A newer alternative, the pulsed electric field (PEF) ablation system, employs a slightly different mechanism than IRE and expands the treatment targets of electrical ablation techniques to include lung cancer. The external energy-based ablation techniques, high-intensity focused ultrasound (HIFU) and histotripsy, have also garnered significant interest given their non-invasive or non-thermal nature.^3^

This review article aims to explore the recent advancements in tumor ablation in IO. We focus on non-thermal electrical and non-invasive ultrasound modalities, specifically their mechanisms and clinical applications. Non-thermal and/or non-invasive ablation techniques are redefining the paradigm of image-guided tumor ablation.

## Exploring electrical options: IRE and PEF

Electrical ablation techniques represent a significant advancement in non-thermal modalities, offering the precision and preservation of surrounding tissues that thermal methods may compromise.^4^^,^^5^ Their main advantage lies in their ability to target cells selectively while minimizing damage to the extracellular matrix, nerves, and blood vessels, which is crucial in treating tumors near vital structures.^1^^,^^2^ The most prominent of these technologies are IRE and PEF, each with distinct mechanisms and applications.

### Mechanism and principles of IRE

**Figure 1.**
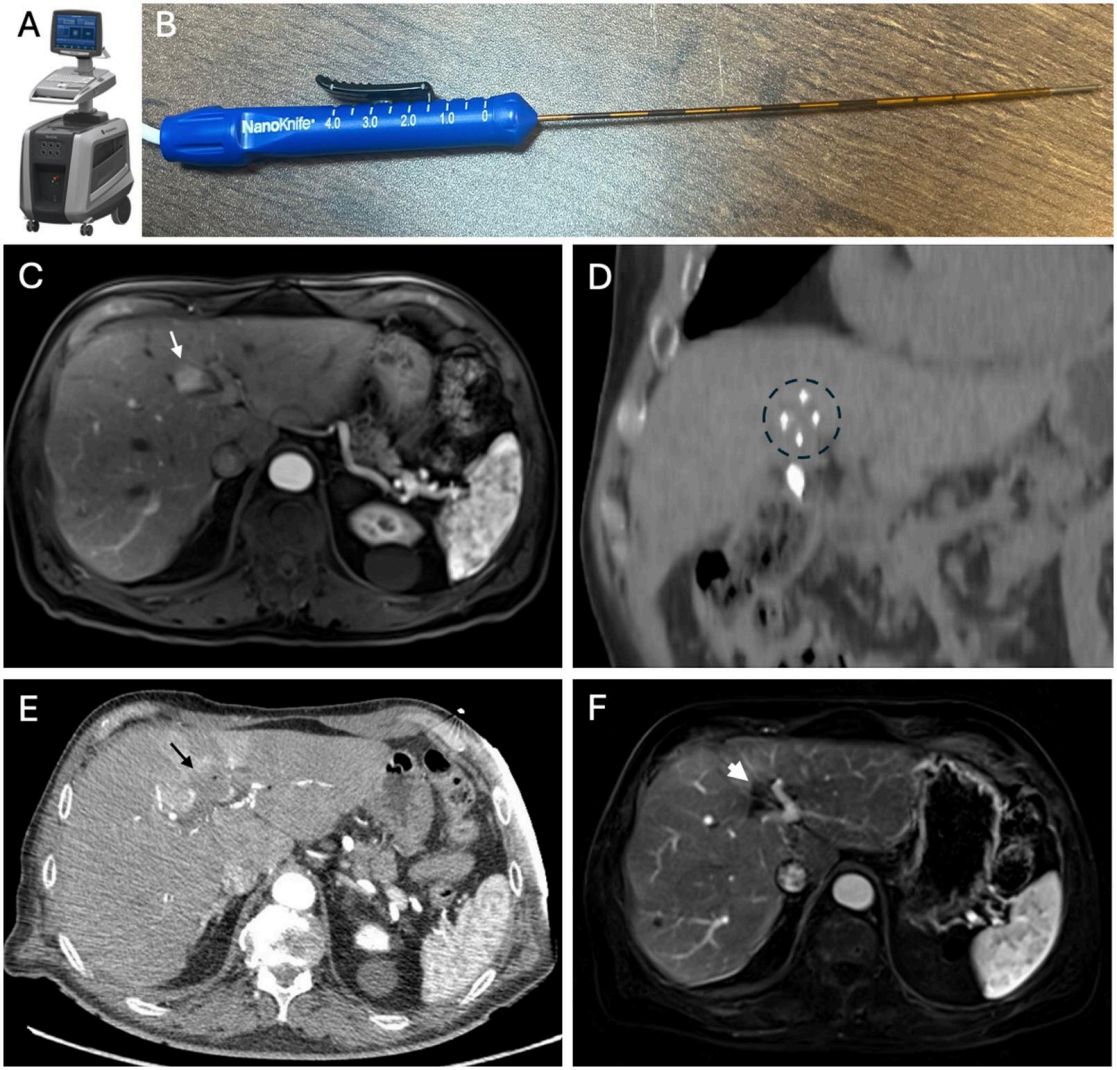
Schematic picture of the irreversible electroporation (IRE) machine and probes, along with a case presentation of IRE in hepatocellular carcinoma (HCC). NanoKnife System (AngioDynamics, Queensbury, NY) displaying the (A) generator and a close-up image of the (B) needle probe. Generator and probe images reprinted with permission from AngioDynamics (Queensbury, NY). IRE was performed in a 72-year-old male with a history of hepatitis C cirrhosis status post-treatment with sustained viral response whose liver demonstrates a lesion not amenable to microwave ablation (MWA) due to close proximity of critical structures: (C) contrast-enhanced MRI demonstrates a 1.7 cm Liver Imaging Reporting and Data System (LI-RADS) 5 lesion (white arrow) in segment IVb; (D) coronal CT image of intra-procedural IRE needles (dotted black circle) placed in parallel position; (E) contrast-enhanced immediate post-procedure CT shows excellent ablation coverage of the LI-RADS 5 lesion (black arrow); (F) contrast-enhanced MRI at 7-month follow-up shows no residual tumor (thick white arrow) nor injury to the biliary system.

IRE is a non-thermal ablation technique that utilizes short-duration, high-voltage electrical pulses between pairs of electrodes to create nanopores in the cell membrane, leading to disruption of the cellular membrane integrity, which causes a loss of cellular homeostasis and eventual cell death via apoptosis.^3^^,^^6^ This contrasts with reversible electroporation, which induces transient nanopores in the cell membrane, allowing cellular membrane integrity to recover without affecting cell viability, and thus is often used to enhance local drug delivery.^2^ IRE is especially valuable in treating tumors located near critical structures, as it has been shown to spare the extracellular matrix of connective tissues and nerve fibers, thus preserving structural integrity.^4^^,^^5^ IRE also minimizes the heat sink effect, making it effective for targeting lesions near large vessels.^1^^,^^2^ Risks of IRE include bleeding, infection, pancreatitis, bile leakage, biliary obstruction, and other complications related to the targeted organ. Currently, the NanoKnife System (AngioDynamics, Queensbury, NY) is the only commercially available IRE treatment system approved by the U.S. Food and Drug Administration (FDA) (Figure 1A and B). Its use is limited by cost and accessibility.

### Clinical applications in cancer therapy

IRE has been successfully applied in the treatment of various cancers, including hepatic, pancreatic, prostatic, and renal cancers, demonstrating potential in cases where traditional thermal ablation or surgical resection is not feasible.^6^

### Liver tumors

IRE may be effective in destroying hepatic tumors close to critical structures (Figure 1C-F), such as the large bile ducts, portal vein, gallbladder, or bowel.^1^^,^^7^ One of the pioneer studies, the COLDFIRE-1 trial, demonstrated irreversible cell injury on pathology in colorectal liver metastases (CRLM) treated with IRE.^8^ The ongoing COLDFIRE-2 trial evaluates the efficacy of IRE for CRLM ≤5 cm that are unsuitable for partial hepatectomy or thermal ablation, with preliminary findings indicating that IRE is both effective and relatively safe.^7^ Other large retrospective studies assessing IRE for the treatment of hepatocellular carcinoma (HCC), cholangiocarcinoma, and CRLM show promising results,^9^ with one study demonstrating similar complication rates between IRE and thermal ablation techniques.^10^ A clinical trial aimed at comparing the safety and efficacy of IRE to RFA for small HCC is currently underway.^11^

### Pancreatic tumors

There are several ongoing clinical trials and multicenter registries evaluating the efficacy and safety of IRE for pancreatic adenocarcinoma, with the most notable being the DIRECT Registry prospective trial involving the treatment of patients with stage 3 pancreatic adenocarcinoma compared to the standard of care (SOC).^12^^,^^13^ All patients received induction chemotherapy. Overall safety data were similar between the IRE and SOC arms, with 30-day mortality rates ranging from 2.3% (IRE) to 3.7% (SOC) and 90-day adverse events ranging from 71.5% (IRE) to 81.5% (SOC).^13^ Grade 3 or higher adverse events ranged from 27.6% (IRE) to 44.4% (SOC).^14^ Overall, IRE in pancreatic cancer shows promising results, especially in combination with neoadjuvant chemotherapy.

### Prostate tumors

Prostate cancer is the most studied oncologic use of IRE. A 2024 review of 14 studies (899 patients) found in-field recurrence rates of 0%-38.9% and out-of-field rates of 3.6%-28% after IRE.^7^ Urinary continence returned to pretreatment levels in 58% of studies, along with overall improvements in erectile function.^15^ Preliminary PRESERVE trial results showed a 67.6% median prostate-specific antigen (PSA) reduction in 74 patients at 6 months, with 8.3% of patients experiencing Grade 3 adverse events and none experiencing Grade ≥4 events.^16^ A 2024 registry of 411 patients found that the International Prostate Symptom Score rose at 3 months but stabilized by 6 months, with adverse events at 1.8% (3 months) and 1% (6 months), remaining stable to 48 months.^17^

### Renal tumors

**Figure 2.**
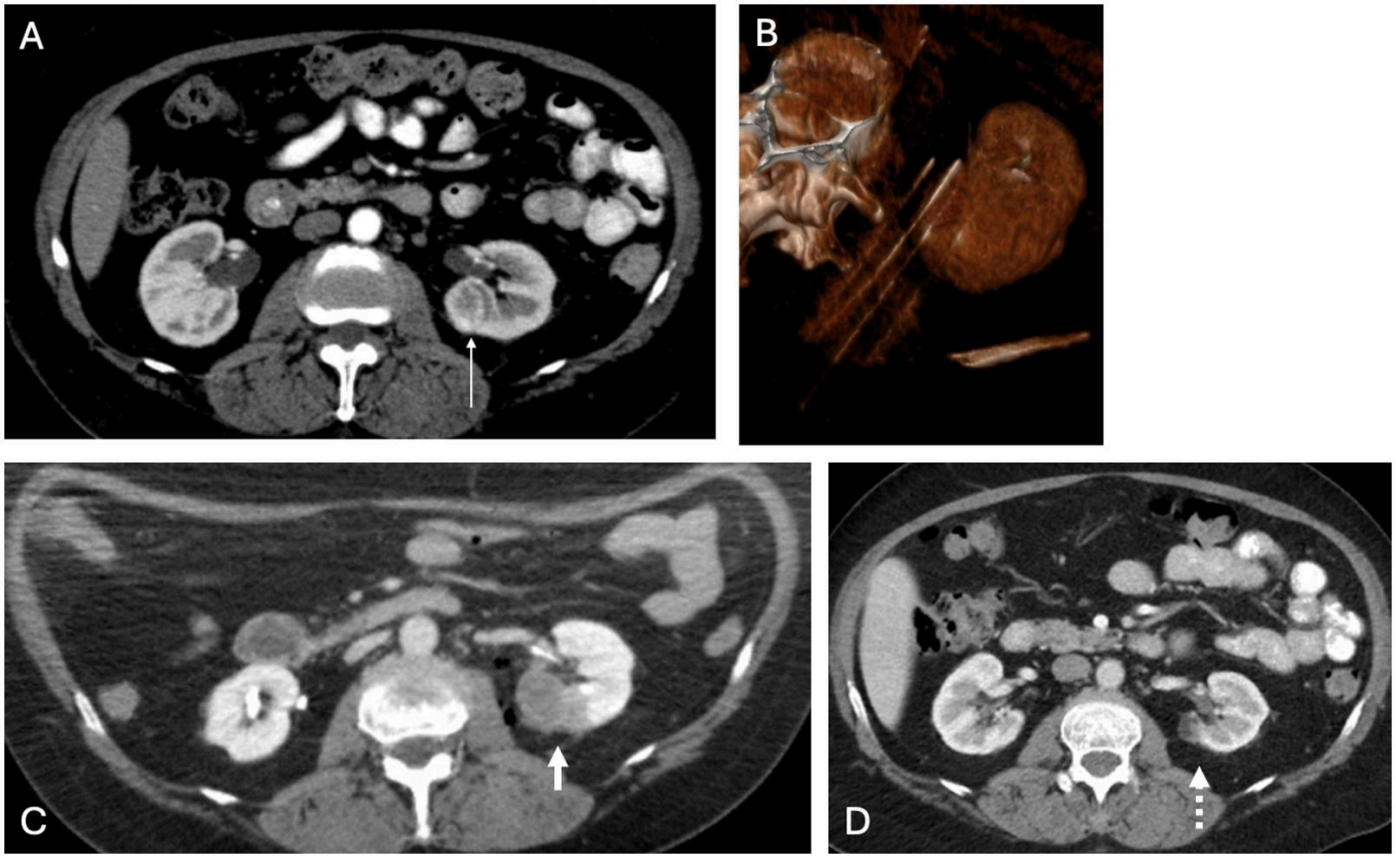
Irreversible electroporation (IRE) in renal cell carcinoma. IRE was performed in a 74-year-old female with left renal cell carcinoma: (A) axial contrast-enhanced CT in the early arterial phase demonstrates a 2.7 cm enhancing mass (white arrow) in the left kidney; (B) maximum intensity projection and 3D reconstructed image demonstrate 4 needles placed in parallel for IRE; (C) tumor enhancement is absent immediately following IRE (thick white arrow); (D) at 4-year follow-up, the tumor remains non-enhancing (dotted white arrow) with no residual tumor or recurrence.

IRE for renal cancer is typically considered when surgery or thermal ablation is contraindicated and when the tumor is near critical structures (Figure 2A-D).^18^ While most of the data have been limited to retrospective studies with small sample sizes, studies have shown promising results.^19^ Several ongoing clinical trials are evaluating the role of IRE in renal cancer treatment.^18^^,^^20^ A 2022 systematic review of 10 studies and 83 patients with renal cell carcinoma found no 30-day mortalities, no significant long-term changes in renal function, and complete response rates of 78% via imaging and 57% via histopathology.^21^

### The emergence of PEF

**Figure 3.**
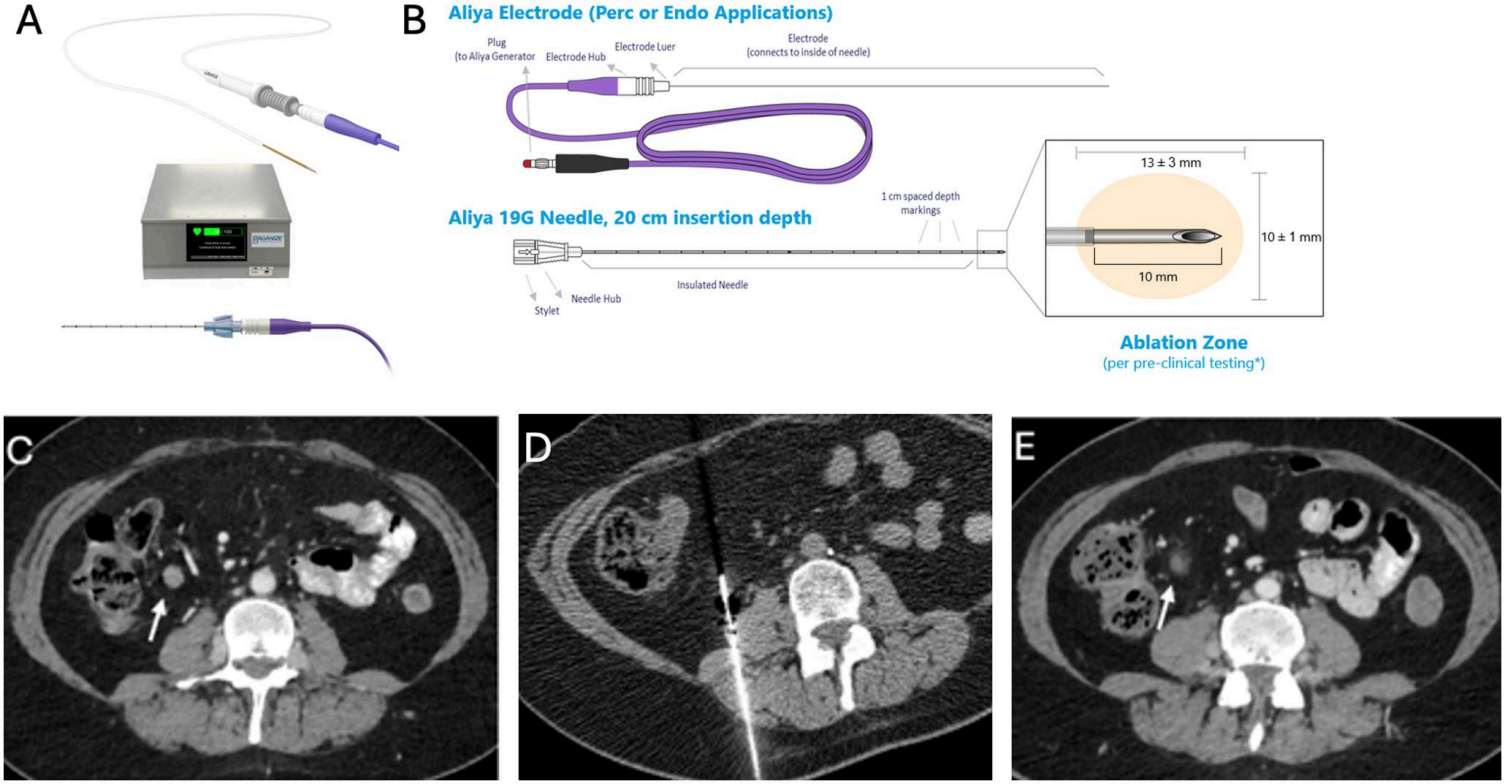
Schematic image of the Aliya System by Galvanize Therapeutics (San Carlos, CA), which offers pulsed electric field (PEF) in a 49-year-old female with a history of endometrial cancer and an enlarging right lower quadrant mesenteric lymph node, suspicious for recurrence. The Aliya System (A) generator and needle/probe with (B) detailed schematic of each of the parts of the needle/probe. Generator and needle/probe schematic images reprinted with permission from Galvanize Therapeutics (San Carlos, CA). (C) Pre-PEF CT scan shows an enlarging right mesenteric lymph node (white arrow) up to 10 mm, suspicious for metastatic disease. (D) PEF procedure: CT-guided biopsy and PEF ablation of a mesenteric right lower quadrant lymph node. (E) Post-PEF CT scan demonstrates an interval decrease in size of the targeted mesenteric lymph node (white arrow) with post-ablation changes, measuring 5 mm in short axis.

PEF is another non-thermal ablation option that utilizes electrical pulses. The Aliya System (Galvanize Therapeutics, GTI-00018 investigational device, San Carlos, CA) is a recently FDA-cleared PEF ablation system that expands electrical ablation to non-small cell lung cancer (Figure 3A and B). Lung cancer is not amenable to conventional IRE, as the energy deposition from IRE probes is deterred by air exposure.^22^ Unlike IRE’s monophasic waveforms generated in between probes, PEF uses biphasic pulses via a single monopolar probe to generate a uniform electric field, resulting in membrane lysis and programmed cell death.^23^^,^^24^ Furthermore, PEF typically employs shorter pulses (nanoseconds vs milliseconds) with a stronger electrical field (300 kV/cm vs 0.1-1 kV/cm), minimizing the risk of temperature increase.^23^ As a result, PEF avoids significant neuromuscular activation and does not require paralysis or general anesthesia. Common risks associated with PEF include pneumothorax, pleural effusion, and bleeding.^24^

Early studies indicate the technical feasibility and safety of PEF in treating primary and metastatic malignancies with mixed oncologic outcomes (Figure 3C-E).^23^^,^^24^ On the one hand, the 2024 retrospective, single-institution study of 17 patients with 30 lesions (90% metastatic, 10% primary) across the thorax (*n *= 20), abdomen (*n *= 7), and head and neck (*n *= 3) found that PEF was well-tolerated and technically feasible.^24^ 50% target lesions and 50% off-target lesions were stable or reduced in size, and 47% of patients had overall stable disease. There were 26 reported adverse events (9 mild, 1 moderate). On the other hand, the 2025 retrospective cohort study of 11 patients with 13 tumors demonstrated a low rate of complete coverage (9%), a high rate of residual, local, or distant progression (91%), and an absent abscopal effect. Adverse events occurred in 36% of cases (2 mild, 2 severe), but there was no adjacent organ injury.^24^

Both IRE and PEF require advanced operator training, including a clear understanding of the electrical energy delivery, ablation zone size and overlap, and optimal electrode/probe placement, to achieve tumor coverage/margin and expected outcomes. Oncologic outcomes and cost-effectiveness remain unclear for both modalities due to limited prospective data with long-term follow-up and higher up-front costs, especially in comparison to the well-established modalities such as RFA and MWA.

The decision to use IRE or PEF over traditional thermal ablation techniques is influenced by operator experience, tumor size, anatomical location, and proximity to critical structures such as bile ducts, bowel loops, or neurovascular bundles. IRE is advantageous for tumors adjacent to large vessels or ducts due to its minimal thermal spread. PEF is suitable for lung ablations and may be appropriate in anatomically challenging regions or patients with high anesthesia risk, given its single-probe approach and minimal muscular activation. Thermal ablation remains SOC for tumors in accessible locations without non-maneuverable critical adjacent structures.

## Exploring non-invasive ultrasound options: HIFU and histotripsy

HIFU and histotripsy are 2 emerging ultrasound-based ablation techniques used in tumor treatment and tissue destruction.^3^^,^^25^ HIFU uses focused ultrasound waves to generate localized thermal energy and induce coagulative necrosis, while histotripsy is a purely mechanical ultrasound-based therapy that creates cavitation bubbles to induce cellular disruption and tissue liquefaction. These techniques offer controlled ablation and structural preservation, which are crucial for treating various tumors.

### Mechanism and principles of HIFU

**Figure 4.**
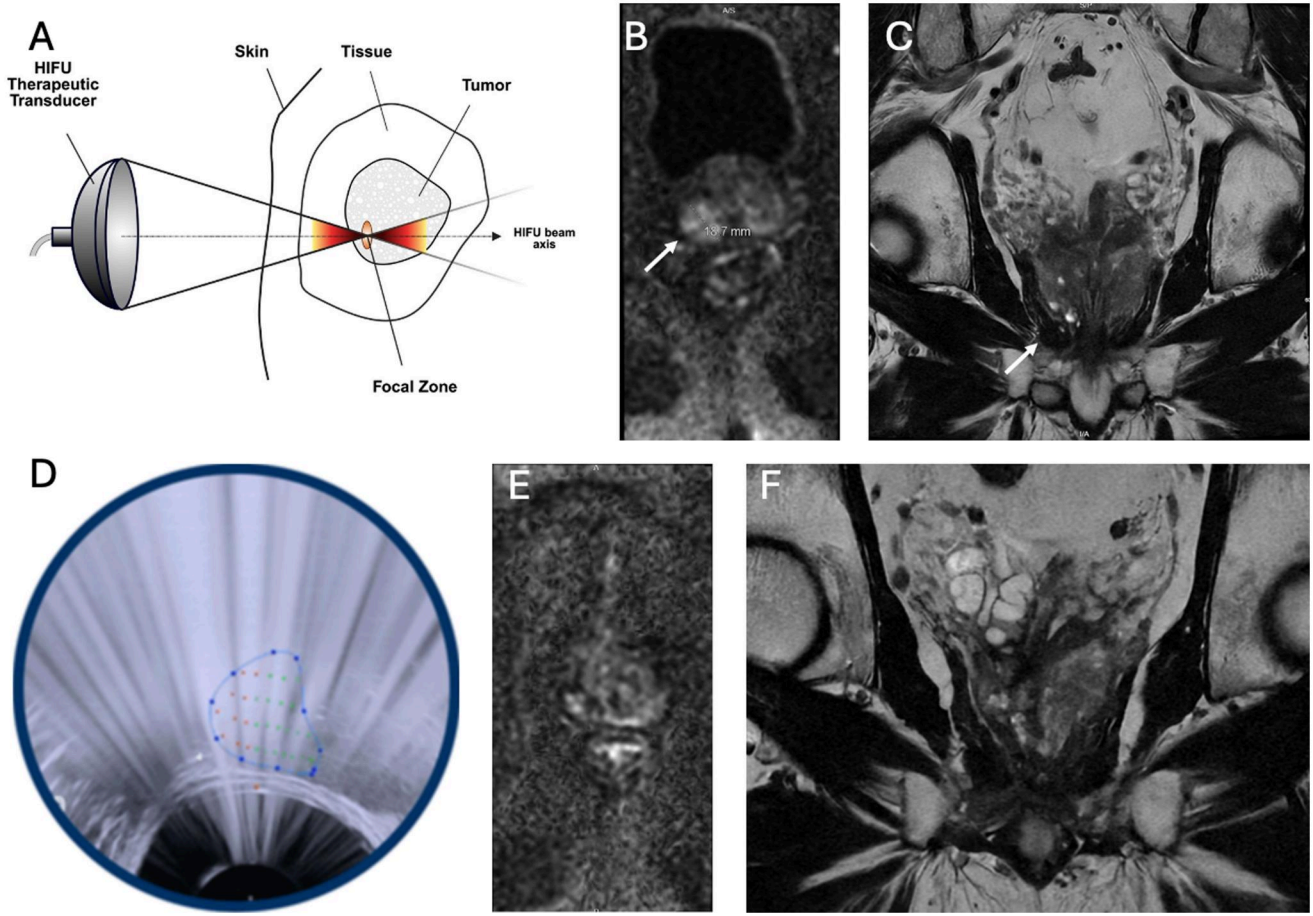
Schematic depiction of high-intensity focused ultrasound (HIFU) ablation and a case of prostate HIFU. (A) The therapeutic transducer emits ultrasound waves that converge on the tumor’s focal zone. HIFU was successfully used to treat a 71-year-old male with prostate cancer with associated PSA 9.4 ng/mL. Pre-HIFU axial diffusion-weighted (B) and coronal T2 (C) MRI images revealed a 1.9 cm Prostate Imaging Reporting and Data System (PI-RADS) 5 lesion in the right posterolateral peripheral zone at the prostate base (white arrow). Thus, robotic HIFU was performed with segmentation and definition of the target area, with (D) real-time ultrasound showing energy delivery to the target contoured by the HIFU (blue dotted line), with treated (orange dots) and not-yet-treated (green dots) locations of the prostate. Post-treatment axial diffusion-weighted (E) and coronal T2 (F) MRI images at 9 months showed no clinically concerning lesions and atrophy of the right prostate lobe at the site of HIFU treatment.

HIFU is a minimally invasive, non-ionizing ablation technique that enables deep tissue penetration (1-20 cm) with minimal surrounding or intervening tissue damage.^26^^,^^27^ Its primary thermal mechanism involves the focal convergence of high-intensity ultrasound waves generated from a piezoelectric transducer, raising tissue temperature to 60-85 °C within seconds, inducing protein denaturation, membrane disruption, and irreversible coagulative necrosis (Figure 4A).^28^ The resulting thermal zones are cigar-shaped, comparable in size to a grain of rice (approximately 10 mm in length and 1-5 mm in diameter), and aligned with the direction of ultrasound wave propagation. Overlapping of multiple sonications is necessary to ensure target lesion coverage and margin.^29^ The extent of thermal tissue damage is influenced by various factors, including target tissue absorption and attenuation properties, exposure time, acoustic reflection, and vascular perfusion within the targeted tissue. Additionally, depth significantly impacts energy delivery, as deeper targets (>10 cm) experience greater acoustic attenuation and less effective energy delivery.^30^ Adjustable HIFU parameters, including ultrasound power, frequency, and transducer (ie, shape, type, and size), must be tailored in each clinical context to ensure safe and effective ablation.^29^

Beyond thermal damage, HIFU also induces mechanical effects via acoustic cavitation, which are either stable (non-inertial) or inertial cavitation.^31^ Stable cavitation disrupts tissue microenvironments through rhythmic microbubble oscillations, while inertial cavitation generates powerful shockwaves and extreme localized temperatures of 2 000-5 000 K, leading to significant subcellular fragmentation. These microbubbles synergistically trap and absorb the ultrasound energy, enhancing heat deposition and the associated thermal treatment effects.^29^^,^^31^

HIFU may be performed in multiple sessions with minimal sedation given its minimally invasive nature and the absence of ionizing radiation.^26^^,^^27^ However, treatment duration, bowel gas, scars, and respiratory motion can lower accuracy and increase thermal injury risks.^28^^,^^31^^,^^32^ In complex cases, general anesthesia and controlled ventilation may be required to ensure precise targeting and stability.^33^ Advances in HIFU aim to refine mechanical tissue disruption, improve precision, and expand therapeutic applications.^34^

### Clinical applications in cancer therapy

HIFU has shown significant potential in treating benign and malignant tumors.^26^ Its delivery systems include extracorporeal transducers for both superficial and deep targets, intracorporeal transducers for transrectal or transurethral approach, and interstitial transducers for surgical implantation to provide localized, long-term therapy.^14^^,^^26^ A study of 1 038 patients with solid tumors treated via extracorporeal HIFU showed reduced tumor blood supply, absent radioisotope uptake, and significant regression without major complications.^35^

### Liver tumors

Ablative treatments are first-line treatments for early-stage HCC. HIFU may serve as an alternative to traditional thermal ablative techniques in the management of small-sized HCC <3 cm not amenable to surgical curative treatments, or as a bridging strategy.^36^ In patients with advanced HCC, HIFU may also alleviate symptoms and improve quality of life.^37^

A study of 100 patients with liver cancer (primary and metastatic) treated with the JC model HIFU system showed coagulative necrosis within the target tumors and either significantly reduced or completely eradicated tumor blood supply, with 86.6% experiencing clinical improvement and no complications.^38^ Combining HIFU with transarterial chemoembolization further enhances efficacy, significantly improving overall survival.^39^ A systematic review of 44 studies found that HIFU combined with transarterial chemoembolization yielded survival benefits comparable to RFA.^40^ A clinical trial evaluating HIFU in 13 patients with CRLM showed 100% objective response rate after 1 HIFU session.^41^ Additionally, HIFU has shown promise in treating hepatobiliary malignancies and HCC with portal vein thrombosis. Studies suggest that HIFU may restore hepatic metabolism, boost immunity, and lower tumor markers such as AFP, CEA, and CA19-9.^32^

Despite its potential, the application of HIFU can be limited by tumor location and size, skin thickness, rib interference, respiratory motion, and nearby sensitive structures. Careful evaluation and personalized treatment planning, ideally in a clinical trial setting, are necessary for optimal outcomes.^31^^,^^37^

### Renal tumors

Although partial nephrectomy remains as the gold standard treatment for small renal masses (≤4 cm), thermal ablation techniques (cryoablation, MWA, and RFA) are suitable curative alternatives.^42^ HIFU, similar to traditional thermal ablation techniques, offers reduced morbidity and renal preservation for both curative and palliative purposes. In contrast to percutaneous ablation, HIFU’s extracorporeal approach reduces bleeding and tumor seeding risks.^2^^,^^37^^,^^42^ However, its current use in kidney tumors is limited by potential skin damage, variable tissue destruction, and anatomical obstacles. Advances in image fusion, motion compensation, and phased array transducers may improve HIFU’s effectiveness in treating kidney tumors in the future.^43^

### Prostate tumors

The primary treatments for localized prostate cancer are radical prostatectomy and radiotherapy, both offering excellent tumor control but often causing side effects. HIFU has emerged as a less invasive alternative for patients with localized prostate cancer, particularly in patients with PSA ≤15-20 ng/mL and prostate volume <40 mL (Figure 4B-F),^44^ and in patients with residual disease following radiation therapy.^45^ The success of HIFU ultimately depends on patient selection, primarily multiparametric MRI and targeted biopsies to ensure the cancer is confined to a specific region of the prostate. Studies have shown that HIFU can achieve favorable oncologic outcomes with minimal functional decline, allowing patients to maintain a high quality of life. Its precision reduces the risks of urinary incontinence and erectile dysfunction. Finally, HIFU does not preclude future treatments in patients with recurrence or progression of disease.^30^

A U.S. study of 100 men with localized prostate cancer found that HIFU preserved continence in all cases with no major complications, and 91% of patients avoided radical treatment after 2 years.^46^ Those with bilateral prostate cancer had a higher recurrence risk. In another study of 33 patients, post-treatment biopsies revealed fibrosis in all cases, necrosis in 73%, and residual cancer in 24%.^47^ Residual tumor was frequently located at the edges or outside of the intended treatment zone. Suggested causes include inadequate probe repositioning during treatment, difficulties with real-time temperature monitoring, anatomical challenges like intraprostatic calcifications, and multifocal cancer, all of which complicate complete tumor coverage and may have increased the risk of residual disease.^47^ A multinational prospective study on focal HIFU therapy showed minimal adverse events, 13.4% treatment failure rate, and uncertain long-term efficacy.^48^ Overall, the most common complications were urinary retention (1%-8.8%), incontinence (10%-49.7%), and erectile dysfunction (20%-77%).^49^

Recent findings from the HIFI study provide compelling evidence supporting the use of HIFU for patients with intermediate-grade, localized, and MRI-visible prostate cancer lesions.^50^ This prospective, non-inferiority, non-randomized trial included 3 328 patients across 46 centers, comparing whole-gland or subtotal HIFU to radical prostatectomy. At the 30-month follow-up, 89.8% of men treated with HIFU remained free from salvage treatment, compared to 86.2% in the radical prostatectomy group, demonstrating medium-term oncological equivalence.^50^ Patients undergoing HIFU experienced lower rates of urinary continence deterioration (29% vs 44%) and less decline in erectile function.^50^ While the safety profiles of both treatments were similar, HIFU was associated with a higher incidence of adverse events, primarily urinary retention, whereas radical prostatectomy was associated with more operative injuries.^50^ These findings suggest that HIFU offers a less invasive treatment option with reduced impact on quality of life, making it an attractive alternative for appropriately selected patients.

### Breast tumors

Breast cancer treatment traditionally includes breast-conserving therapy and mastectomy, both showing similar survival rates, with breast-conserving therapy as the gold standard for localized cancer.^51^ Recently, HIFU and cryoablation have gained interest for the treatment of early-stage breast cancer, offering psychological and cosmetic benefits.^52^ HIFU has been successfully used for various breast cancer subtypes, including invasive lobular and ductal carcinoma.^26^^,^^52^ A systematic review of HIFU therapy for primary breast cancer, analyzing nine studies, reported variable tumor coagulation necrosis rates (17%-100%) and favorable safety, with no severe complications and mild side effects like pain, edema, and burns.^52–54^ Additionally, 2 randomized controlled trials showed a 95% 5-year disease-free survival rate.^53^^,^^54^ However, HIFU’s inability to assess margin status and risk of complications such as skin burns, necrosis, seroma, and ecchymosis limit its current use.^28^^,^^52^^,^^53^

### The emergence of histotripsy

HIFU offers distinct advantages over traditional ablation by eliminating the need for transcutaneous probes and enabling repeat treatments without cumulative radiation exposure.^27^ However, its primary thermal mechanism and the associated risks of thermal energy sink effects and unintended thermal injury hinder its wider clinical adoption.^28–32^ This has led to a growing interest in extracorporeal ultrasound therapies that can achieve mechanical tissue disruption without thermal coagulation.^34^^,^^55^

Histotripsy is an emerging focused ultrasound ablative technique that is non-invasive, non-ionizing, and non-thermal. It mechanically liquefies the targeted tissue using high-amplitude ultrasound pulses, avoiding thermal energy sink effects and thermal injury to adjacent critical structures.^14^^,^^56^ Limitations include depth-related ultrasound attenuation or artifact from overlying structures. Despite lower imaging costs and easier accessibility compared to MRI-guided HIFU,^25^ the high initial capital expense of a histotripsy system may be a barrier to entry.

### Mechanism and principles of histotripsy

Histotripsy directs high-intensity ultrasound pulses, lasting from microseconds to milliseconds, at a low-duty cycle to induce cavitation and subsequent bubble collapse, leading to tissue liquefaction and mechanical disintegration.^29^ On ultrasound, the bubbles appear hyperechoic and create a hypoechoic cavity.^14^ The body absorbs the liquefied tissue, resulting in minimal scarring over a period of 1-2 months.^14^^,^^56^

Histotripsy methods can be further categorized into cavitational, boiling, and hybrid mechanisms. Cavitational histotripsy encompasses shock-scattering and intrinsic threshold (microtripsy) techniques, differing in pulse duration and cavitation thresholds.^55^ Shock-scattering cavitational histotripsy uses microsecond pulses at sub-threshold pressures to expand incidental microbubbles into dense cavitation clouds, while microtripsy employs fewer cycles at higher peak negative pressures to exceed the intrinsic cavitation threshold of the tissue, which subsequently leads to the growth and collapse of cavitation bubbles that result in precise mechanical disruption and liquefication of the target tissue.^57^^,^^58^ Boiling histotripsy employs millisecond pulses to generate vapor bubbles, leading to mechanical tissue fractionation and acoustic atomization, which is the high-velocity ejection of micrometer-sized particles, without significant thermal damage due to lower peak pressures and longer interpulse intervals compared to cavitational histotripsy.^59^^,^^60^ The ablation zone initially appears oblong but evolves into a tadpole shape as treatment progresses. Both cavitational histotripsy and boiling histotripsy provide sharp ablation margins and tissue selectivity.^59^ Hybrid histotripsy is the newest histotripsy method, initially reported in 2016.^61^ Hybrid histotripsy integrates shock-scattering and boiling histotripsy by utilizing pressure amplitudes between the 2, along with pulse lengths lasting submilliseconds. Unlike boiling histotripsy, boiling temperature is not reached with each pulse, but mild heating softens the tissue, enhancing its susceptibility to cavitation and shock-scattering.^55^ Further comprehensive studies and clinical trials are needed to confirm margin adequacy and evaluate the full potential of these histotripsy techniques, ideally through comparisons with established ablation techniques such as MWA in the liver.^62^

### Clinical applications in cancer therapy

Histotripsy has been researched for various types of diseases, most of which were preclinical. Until now, there have been few clinical trials, all of which used shock-scattering cavitational histotripsy. FDA’s approval of the Edison System (HistoSonics, Inc., Ann Arbor, MI) for liver tumor histotripsy marks a significant milestone. Ongoing clinical trials for kidney cancer and pancreatic cancer will likely lead to additional clinical indications in the near future.^63^

### Liver tumors

**Figure 5.**
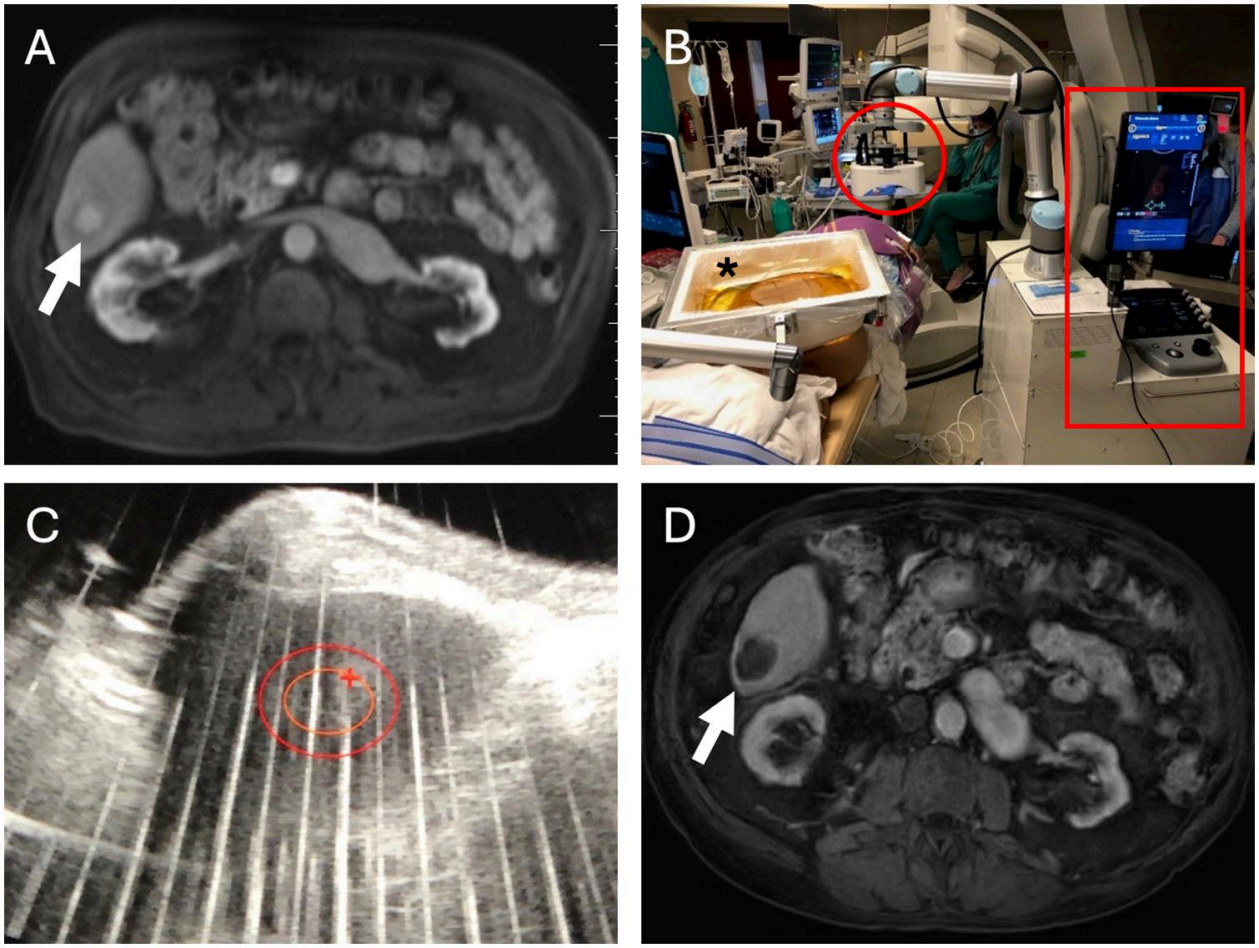
Histotripsy for primary liver cancer. 72-year-old male with alcoholic cirrhosis with 2.1 cm hepatocellular carcinoma: (A) axial contrast-enhanced MRI shows a 2.1 cm arterially enhancing lesion (arrow) in segment VI; (B) the patient was treated with the Edison System (HistoSonics, Inc.), which includes the probes (circle), computer unit (rectangle), and a water bath (*) placed over the patient’s abdomen, over which the system delivers pulsed sound energy to the hepatic tumor; (C) ultrasound imaging demonstrates the sound energy pulses during treatment of the target tumor (orange oval), centered within the total ablation zone (red oval); (D) 1-month post-treatment contrast-enhanced MRI demonstrates complete response with no enhancement in the treated area (arrow).

Histotripsy can be used to treat both cancerous and benign liver tumors (Figure 5A-D). In 2019, a phase I trial in Barcelona treated 11 unresectable liver tumors (including metastases and HCC), with 71.8% average volume reduction after 2 months.^64^ One tumor was mistargeted due to imaging limitations, but no device-related complications occurred. A recent multicenter trial in the United States and Europe, #HOPEFORLIVER, involving 44 patients with 49 tumors, showed 95% technical success rate, with FDA approval now granted for liver tumor treatment.^65^

### Renal tumors

Animal models have shown that histotripsy effectively treats renal cell carcinoma, particularly in the Eker rat.^66^ Boiling histotripsy ablation has been shown to be safe in animal models, sparing the collecting system and preserving adjacent urothelium.^65^^,^^66^ Following these results, human trials are now underway, including the FDA-approved #HOPE4KIDNEY trial, which treated its first patient on January 10, 2024.^63^

### Prostate tumors

The first Phase I human trial on histotripsy for benign prostatic hyperplasia was conducted in the United States in 2016-2017.^67^ Ultrasound pulses delivered transperineally demonstrated that prostate histotripsy is safe, well-tolerated, and associated with significant improvements in the International Prostate Symptom Score. However, unlike preclinical canine studies that showed effective tissue debulking and liquefaction, this trial did not achieve tissue destruction, possibly due to inadequate ultrasound pressure or pulse numbers. Recent studies, however, have demonstrated complete fractionation of targeted ex vivo human prostate tissues and tumors using boiling histotripsy.^56^

### Other potential tumors

Numerous animal studies have explored histotripsy methods for brain, skin, pancreatic, and breast cancers, as well as sarcoma and lymphoma, demonstrating improved survival, decelerated tumor growth, systemic antitumor immune response, and histotripsy-induced abscopal effect.^14^^,^^68^

## Combination treatment: ablation with immunotherapy

All ablative therapies induce immunogenic cell death, releasing tumor-associated antigens and damage-associated molecular patterns, which activate antigen-presenting cells and subsequently, cytotoxic T lymphocytes.^68^^,^^69^ The newly activated cytotoxic T lymphocytes proliferate and induce apoptotic cascades in cancer cells, culminating in a systemic antitumor response. Combination therapies integrating non-thermal ablation with immune checkpoint inhibitors, such as anti-CTLA-4 or anti-PD-L1, have shown promising results in preclinical models, including enhanced survival, untreated tumor regression (abscopal effect), and long-term immune memory.^69^ For instance, in mouse neuroblastoma models, combining both anti-CTLA-4 and anti-PD-L1 checkpoint inhibitors with HIFU yielded 62.5% survival over 300 days and complete regression of untreated tumors compared to 0% survival with control or single therapy and 11% survival with both checkpoint inhibitors without HIFU.^69^ This triple combination therapy also induced durable antitumor immune memory, as evidenced by resistance to tumor formation when treated with double the tumor cell load.^69^ However, such systemic antitumor immune responses and abscopal effects have not been successfully reproduced in human patients in large studies. Until the widespread availability of trial-validated data, the preclinical data should be interpreted and applied with caution.

## Future directions

As non-thermal and/or non-invasive ablation techniques transition from preclinical animal models to early clinical applications as their mechanisms (Table 1) and clinical applications (Table 2) become better understood, emphasis must be placed on data-driven practice, standardized treatment protocols, and patient-centered outcomes. Many of the included studies in this review lack clear inclusion criteria, sufficient power, a generalizable and reproducible treatment protocol, or systematic post-procedural follow-up. A 2021 study in non-metastatic pancreatic cancer patients noted significant post-IRE declines in quality of life and increased pain, highlighting the importance of evaluating post-procedural outcomes and deciphering prognostic factors to facilitate patient selection.^70^ The optimal timing and sequence of ablative and immunotherapeutic modalities also remain largely unexplored. Other practical considerations include barriers to entry, cost-effectiveness compared to SOC, and a system for disseminating proceduralist training and experience, which may limit widespread use.

**Table 1.** Principles and technical considerations of the emerging ablation techniques.

Technique	Principles of mechanism	Pros	Cons	Machines/vendors	FDA approval status
IRE	Permanent nanopore formation in cell membranes, inducing apoptosis^3^^,^^6^	Non-thermal Preserves extracellular matrix and critical structures^4^^,^^5^	Requires general anesthesia, paralytics, and cardiac synchronization^1^ Risk of muscle contraction^1^ Requires multiple probes with precise spacing intervals	NanoKnife (AngioDynamics)	FDA cleared for soft tissue ablation
PEF	Short, high-voltage electric pulses cause pore formation in the cell membrane, inducing apoptosis^23^	Non-thermal No paralysis or general anesthesia needed Single probe^23^^,^^24^	Small ablation zone requiring multiple ablation stations and probe repositioning Requires cardiac synchronization Higher up-front costs	Aliya (Galvanize Therapeutics)	FDA cleared for soft tissue ablation; clinical trials ongoing for cancer-specific indications, such as treating stage IV NSCLC or lung metastases
HIFU	Focused acoustic energy causes mechanical or thermal tissue disruption^28^	Extracorporeal energy delivery without intervening tissue penetration^14^^,^^28^^,^^34^ Non-ionizing^3^	Limited by the acoustic window Near-field heating Long procedure times Skin complications (ie, skin burns, blisters/ulcers, erythema, edema, skin necrosis, hyperpigmentation)^28^^,^^52^^,^^53^	ExAblate (Insightec); Sonablate (SonaCare)	FDA approved (prostate, fibroids, bone metastases)
Histotripsy	Acoustic cavitation from high-intensity ultrasound pulses causes tissue fractionation and mechanical disintegration^14^^,^^29^	Extracorporeal energy delivery without intervening tissue penetration Non-ionizing^3^ Non-thermal^3^ Real-time imaging integration	Limited by the acoustic window Least widely available, newest, and least studied Low cavitation threshold^57–60^ Depth-related ultrasound attenuation artifact	Edison (HistoSonics)	FDA Breakthrough Devices Program; clinical trials (prostate, liver)

Abbreviations: FDA = U.S. Food and Drug Administration; HIFU = high-intensity focused ultrasound; IRE = irreversible electroporation; NSCLC = non-small cell lung cancer; PEF = pulsed electric field.

**Table 2.** Practical considerations for implementing the emerging ablation techniques.

Technique	Tumor/target organs	Size/location considerations	Sedation requirements
IRE	Liver, pancreas, kidney, prostate	≤3-5 cm; best near vessels or ducts Image-guided needle placement required	General anesthesia with neuromuscular blockade^1^
PEF	Liver, pancreas, breast, lung	Ideal for small lesions ≤2 cm	Moderate sedation Deep sedation General anesthesia
HIFU	Prostate, uterus, bone, pancreas, breast	Requires a clear acoustic window Superficial tumors preferred^30^	No sedation Light to moderate sedation Deep sedation General anesthesia
Histotripsy	Liver, kidney, prostate	Optimal for soft tissue and near-vessel lesions ≤3 cm	Light to moderate sedation Deep sedation General anesthesia

Abbreviations: HIFU = high-intensity focused ultrasound; IRE = irreversible electroporation; PEF = pulsed electric field.

## Conclusion

Electrical and ultrasound ablative modalities are emerging as promising modalities to reshape the field of IO. These innovative techniques offer significant therapeutic potential while diminishing risks associated with traditional thermal ablation techniques. Their non-invasive and/or non-thermal nature of cellular destruction, and synergistic effects when combined with systemic treatments, may enhance the efficacy of tumor ablation and expand the scope of image-guided oncologic interventions.

## Supplementary Material

umaf033_Supplementary_Data

## Data Availability

No new data were generated or analyzed in support of this research.
