# Supplementary material for: The growing armamentarium of image-guided tumor ablation in interventional oncology
Source: Radiol Adv. 2025 Sep 11;2(5):umaf033. doi: 10.1093/radadv/umaf033 (PMC12483156; doi:10.1093/radadv/umaf033)
Supplement: umaf033_Supplementary_Data [file umaf033_supplementary_data.zip › 539566811_130623444_1755193377950.pdf]

ICMJE DISCLOSURE FORM

Instructions

In the interest of transparency, we ask you to disclose all employment/relationships/activities/interests listed below that are related to the content of your manuscript. “Related” means any relationship with for-profit or not-for-profit third parties whose interests may be affected by the content of the manuscript. Disclosure represents a commitment to transparency and does not necessarily indicate a bias. If you are in doubt about whether to list an employment/relationship/activity/interest, it is preferable that you do so.

The following questions apply to your employment/relationships/activities/interests as they relate to the **current manuscript only**. Each author is required to submit a separate form and is responsible for the accuracy and completeness of the submitted information.

Your employment/relationships/activities/interests should be **defined broadly**. For example, if your manuscript pertains to the epidemiology of hypertension, you should declare all relationships with manufacturers of antihypertensive medication, even if that medication is not mentioned in the manuscript.

Date: 27-Jun-2025

req First Name: Sanjeeva

req Last Name: Kalva

Manuscript Title: The Growing Armamentarium of Image-Guided Tumor Ablation in Interventional Oncology

Manuscript number: RADADV-2025-035.R3

In item #1 below, report all support for the work reported in this manuscript without time limit. For all other items, the time frame for disclosure is the past 36 months. Note: All items #1 through #13 must indicate none (by checking the box next to None) or include relevant disclosure information in the text boxes. Blank rows will cause the form to be sent back for completion.

	**Name all entities with whom you have this relationship or check the box next to None**	**Specifications/Comments (e.g., if payments were made to you or to your institution)**
**Time frame: Since the initial planning of the work**		
1. All support for the present manuscript (e.g., funding, provision of study materials, medical writing, article processing charges, etc.) **No time limit for this item.**	✓	None

**Time frame: past 36 months**		
2. Grants or contracts from any entity (if not indicated in item #1 above).	☐	None
	Research grants to the institution: CRICO, NIH, BD, Trisalus, Instylla, Medtronic, FluidX Medical, SIRTEX, Black Swan,	Payment to the institution
3. Royalties or licenses	☐	None
	Elsevier, Springer	Paid to me.
4. Consulting fees	☐	None
	Medtronic, Boston Scientific, CR group, S2N Health LLC, Okami, BD, Canon	Payments to me
5. Payment or honoraria for lectures, presentations, speakers bureaus, manuscript writing or educational events	☐	None
	Apollo Radiology International, Medtronic, Canon Medical, Institute of Liver and Biliary Sciences, India, Liver Foundation, India, Boston Scientific,	Payments to me
6. Payment for expert testimony	✓	None
7. Support for attending meetings and/or travel	☐	None
	Apollo Radiology International, Medtronic, Canon Medical, Institute of Liver and Biliary Sciences, India, Liver Foundation, India,	Paid to me
8. Patents planned, issued or pending	✓	None

9. Participation on a Data Safety Monitoring Board or Advisory Board	✓	None
10. Leadership or fiduciary role in other board, society, committee or advocacy group, paid or unpaid	☐	None
	Chair of Vascular Panel, Appropriateness Committee for the American College of Radiology International Division Councilor, Society of Interventional Radiology Assistant Editor/Consultant to Editor, Radiology – Cardiothoracic, RSNA International Editor, Journal of Clinical Interventional Radiology ISVIR Chief, Interventional Radiology, Massachusetts General Hospital, Boston, MA Vice Chair of Image Guided Interventions, Department of Radiology, University of Texas Southwestern Medical Center, Dallas	Me
11. Stock or stock options	☐	None
	Adobe, AMD, Affirm holdings, Airbnb, Alibaba, Allakos, Allogene Therapeutics, Alphabet,	Me and spouse

	Amazon, AMC, Apple, ARM, Baidu, Best Buy, Beyond Meat, Big lots, Block Inc, Boeing, Cassava sciences, Chemours Co, Children's Pl, Chipotle, Coinbase, Datasea, Docusign, Doximity, FedEx, Fiverr Intl, Gap, GM, Google, Infinity pharmaceuticals, JX Luxventure, Kohl's, Lucid, Lululemon, Macy's, Meta, Moderna, Netflix, Nice, Nikola, Neo, Nvidia, Okta, Pala Alto networks, Paypal, Paysafe, PDd holdings, Qualcomm, Rivian automotive, Roku, Salesforce, Shopify, Snowflake, Sofi technologies, Super micro computer Inc, Tesla Inc, Trade desk inc, Twilio Inc, Under Armour, Upexi Inc, Upstart Holdings, Victoira secrets, Virgin Galactic Holdings, Walt Disney company, Wayfair Inc, Zoom video communications, Biogen Inc, Clover Health Investments Corp, Inovio Pharmaceuticals, Pfizer Inc, Novavax Inc,	

	Orphazyme, Vivos Therapeutics, Ardelyx Inc, Althea Health, Sarepta Therapeutics, Clover Health Invetments Corp, CureVac BV, Immunoprecise antibodies ltd, Zymergen Inc, BioNTech SE, Trillium Therapeutics, Theravance Biopharma Inc, Ergo Inc, Allogent Therapeutics Inc, NRx Pharmaceuticals Inc, Atea Pharmaceuticals Inc, Fate Therapeutics, Vespera Care	
12. Receipt of equipment, materials, drugs, medical writing, gifts or other services	✓	None
13. Other financial or non-financial interests	☐	None
	Adjunct faculty, Christian Medical College, Vellore, India<br>Advisor, KMCH Institute of Health Sciences, Coimbatore, India<br>Professor of Radiology, Harvard Medical School<br>Professor of Radiology, UT Southwestern Medical center<br>Visiting Professor, Christian Medical College, Vellore, India	

req **Please check the box next to the following statement to indicate your agreement:**

✓ I certify that I have answered every question and all the information is complete and accurate.

This is a reprint of the ICMJE Recommendations for the Conduct, Reporting, Editing and Publication of Scholarly Work in Medical Journals. RSNA prepared this reprint. The ICMJE has not endorsed nor approved the contents of this reprint. The official version of the Recommendations for the Conduct, Reporting, Editing and Publication of Scholarly Work in Medical Journals is located at www.ICMJE.org. Users should cite this official version when citing the document.
